# Positive Emotions and Entrepreneurial Intention: The Mediating Role of Entrepreneurial Cognition

**DOI:** 10.3389/fpsyg.2021.760328

**Published:** 2021-11-12

**Authors:** Ben-Song Chen, Chih-Hung Yuan, Bin Yin, Xiao-Zhi Wu

**Affiliations:** School of Economics and Commerce, Zhongshan Institute, University of Electronic Science and Technology of China, Zhongshan, China

**Keywords:** positive emotions, entrepreneurial cognition, arrangement scripts, willing scripts, ability scripts, entrepreneurial intention, PLS-SEM

## Abstract

Under the background of “mass entrepreneurship and innovation,” entrepreneurship and innovation for college students not only alleviates the current social employment pressure but also sets off the upsurge of their entrepreneurship. It is a significant field to research the entrepreneurial intention of undergraduates as potential entrepreneurs, which covers the study of entrepreneurial intention from the perspective of personal traits and entrepreneurial cognition. This article studies entrepreneurial intention from two aspects: irrational positive emotions and rational entrepreneurial cognition, which aims to reveal the mechanism of positive emotions and entrepreneurial cognition on entrepreneurial intention. After investigating 288 college students participating in entrepreneurial competitions, establishing structural equations, and using SmartPLS software for data analysis, the research result showed that positive emotions significantly positively impact the three scripts of entrepreneurial cognition: arrangement scripts, willing scripts, and ability scripts. The arrangement, willing, and ability scripts positively influence entrepreneurial intention, while positive emotions do not affect entrepreneurial intention. Arrangement scripts and ability scripts have a full mediating effect between positive emotions and entrepreneurial intention. Based on these findings, we provide suggestions for the government and society, schools, and individual students on innovation and entrepreneurship.

## Introduction

In September 2014, Premier Li Keqiang put forward the concept of “Mass entrepreneurship and innovation.” Since then, China has set off a new wave of “Mass entrepreneurship” and “Grass-root entrepreneurship” from top to bottom, forming a unique situation of “Mass innovation” and “Everyone innovation.” Youth is the leading force in entrepreneurship, and the Chinese government actively encourages college students to innovate and start their businesses.

In August 2012, the Ministry of Education of China formulated the “Basic Requirements for Entrepreneurship Education in General Undergraduate Schools (Trial),” colleges and universities required to offer a separate compulsory course of “Entrepreneurship Fundamentals” for all students to teach entrepreneurial knowledge, to exercise entrepreneurial capabilities, and cultivate an entrepreneurial spirit. At the same time, to encourage college students to practice entrepreneurship and innovation, the Ministry of Education led the launch of the “Internet +” college student innovation and entrepreneurship competition and the national college student e-commerce “Innovation, Creativity and Entrepreneurship” challenge (after this referred to as the “3chuang”). The scope involves nearly 20 million people across the country. However, according to the “Bluebook of employment: Chinese 4-year college graduates’ employment annual report (2020)” (Wang and Chen, 2020), In 2019, the proportion of self-employed graduates is 1.6%, while the proportion of self-employed higher vocational graduates is 3.4%. Thus, although the government encourages college students to start their businesses efficiently, the actual ratio of college students’ entrepreneurship is still low.

Potential entrepreneurs refer to people who may start a business in the future. Potential entrepreneurs are like the “good seeds” of entrepreneurship, which will germinate and blossom when the time is ripe. College students are naturally the “good seeds” – potential entrepreneurs who are more innovative, proactive, and exhibit a higher risk tolerance (Bolton and Lane, 2012). Research on them is usually related to entrepreneurial intentions (Esfandiar et al., 2019).

Entrepreneurial intention is defined as a focused mentality that guides personal attention and experience toward planned entrepreneurial behavior (Do and Dadvari, 2017). According to the Theory of Planned Behavior, the entrepreneurial behavior of college students is affected by the entrepreneurial intention, and the entrepreneurial intention is affected by the attitude, subjective norms, and perceived behavior control. From the social cognition theory, entrepreneurship is considered a conscious, planned behavior guided by behavioral willingness (Krueger et al., 2000). Individual entrepreneurial intention is an effective predictor of entrepreneurial behavior (Liñán et al., 2011). However, the factors affecting an undergraduate’s entrepreneurial intention are very complex, including individual characteristics, family factors, school factors, and social factors, which makes it difficult for people to understand the formation mechanism of college students’ entrepreneurial intention.

Mitchell et al. (2002) started from the two concepts of entrepreneurship and cognition and believed that entrepreneurship cognition should concentrate on how entrepreneurs interpret, analyze, store, and use entrepreneurial-related information in the market environment. First, the research focused on entrepreneurs’ knowledge structure and information processing process on entrepreneurial opportunity identification, enterprise establishment, and growth. Then, researchers began with the knowledge structure and information processing methods of entrepreneurs to deeply analyze their behavior mechanism and better answered the three questions that entrepreneurship researchers have been paying attention to for a long time: that is, why some people want to become entrepreneurs, why some people can identify entrepreneurial opportunities, and why some people are more successful in entrepreneurship.

Entrepreneurship intention is a subjective attitude of potential entrepreneurs on whether to engage in entrepreneurial activities, and entrepreneurial intention is an integral part of entrepreneurial practice activities. However, existing scholars mainly analyze the influencing factors of entrepreneurial intention from a rational perspective and pay less attention to irrational factors. Emotions have an important impact on entrepreneurial intention (Cardon et al., 2013), and entrepreneurial cognition can show the thinking process of entrepreneurs when making decisions and discern the rational laws of entrepreneurial activities.

Given this, this research will explore the following questions. First, how does entrepreneurial cognition as a rational factor affect the entrepreneurial intention of potential entrepreneurs? Second moreover, how does positive emotion as an irrational factor affect the entrepreneurial intention of potential entrepreneurs? Finally, how do entrepreneurial cognition and emotions work together on the entrepreneurial intention of potential entrepreneurs? This article is expected to theoretically reveal the mechanism of positive emotions, entrepreneurial cognition, and entrepreneurial intention of college students as a potential entrepreneurial group and provide suggestions for government and society, schools, and individual students at the practical level of innovation and entrepreneurship.

We selected a specific group of students who participated in the “3chuang” Challenge as the survey object to clarify the above issues. This group has a young, school, professional background, and basic knowledge of entrepreneurship. Moreover, there is the entrepreneurial practice of entrepreneurial competition. Therefore, through the investigation of this group and establishing a structural equations model for analysis, the influence mechanism of positive emotions, entrepreneurial cognition, and entrepreneurial intention may be derived to provide a realistic reference for entrepreneurship education and entrepreneurship policies of schools, governments, and society.

Subsequent chapters of the paper first reviewed the relevant literature related to the theoretical foundation proposed the research hypothesis, materials and methods, carried out statistical analysis through PLS-SEM, reported the research results, discussed, and finally drew the conclusion and points out the research significance.

## Literature Review

In order to reveal the mechanism of irrational positive emotions and rational entrepreneurial cognition on entrepreneurial intention, this article reviewed relevant literatures on positive emotions, entrepreneurial cognition, and entrepreneurial intention, laying a theoretical foundation for this study.

### Entrepreneurial Intention

The term entrepreneurial intention comes from the concept of intention in social psychology. Intention reflects the individual’s belief in a particular social behavior in the future and the specific psychological state resulting from it. Therefore, entrepreneurial intention refers to the psychological state of an individual to engage in entrepreneurial activities, including attention, expectation, consideration, commitment, and belief (Gupta and Bhawe, 2007; Yuan et al., 2020). Some researchers define entrepreneurial intention as some behaviors of individuals before the formal implementation of entrepreneurship, such as planning, preparation, and effort (Liñán and Chen, 2010; Sesen, 2013; Vinogradov et al., 2013; Yao et al., 2016). Thus, call it the “pre-behavior” of entrepreneurship. However, defining entrepreneurial intention as the “pre-behavior” of entrepreneurship is beyond the psychological concept of “intention” scope, so it is not entirely appropriate. Other researchers define entrepreneurial intention as the willingness to “start a new company” (Zhao et al., 2005; Souitaris et al., 2007), and the willingness to “self-employ” (Lüthje and Franke, 2003; Douglas and Shepherd, 2014), the willingness to “start your own business” (Fayolle et al., 2006), and the willingness to “develop new value elements within the existing organization” (Wu and Wu, 2008). This kind of definition has the problem of generalizing entrepreneurial intention to a certain extent. This study believes that entrepreneurial intention mainly refers to an individual’s psychological tendency to start a new business.

Regarding the measurement of entrepreneurial intention, the measurement methods and the structural dimensions of the measures adopted by the researchers are quite different. Some researchers use single-variable measurement methods to measure individual entrepreneurial preferences (self-employment), entrepreneurial expectations (whether they want to start a business), and entrepreneurial possibility. For example, Krueger et al. (2000) have used the topic “Estimate your chances of starting a company in the next 5years” to measure individual entrepreneurial intention. However, most researchers use the multi-variable measurement method due to the significant measurement error in the single-variable measurement method. For example, Liñán and Chen (2010) used six questions, such as “I am ready to do anything for entrepreneurship” to measure individual entrepreneurial intention.

### Entrepreneurial Cognition

Cognition in cognitive psychology mainly includes the information processing process: memory, perception, thinking (concept, reasoning, and creativity/problem solving), and information representation form: representation and speech (illustration and cognitive map; Neisser, 2014). Entrepreneurial cognition is proposed by placing general cognition under the context of entrepreneurship. Baron (1998) believes that entrepreneurial cognition is the main difference between entrepreneurs and non-entrepreneurs, including counterfactual thinking, attribution styles, and planning fallacies. By reviewing the definitions of “entrepreneurship” and “cognition,” Mitchell et al. (2002) further defined entrepreneurial cognition as the knowledge structure for entrepreneurs to evaluate, judge, and make decisions on critical entrepreneurial issues, such as business opportunities, venture capital, and enterprise growth. Entrepreneurship cognition is the process by which entrepreneurs use a simplified thinking model when making decisions about new products or new services, using various information and resources to discover business opportunities and achieve profit growth (Busenitz et al., 2003). Although different researchers have put forward their definitions of entrepreneurial cognition, interpretation of entrepreneurial cognition of Mitchell et al. (2002) has been widely recognized because it reveals the essence of entrepreneurial cognition and is scientific, accurate, and authoritative.

The research of Mitchell et al. (2002) was the milestone and turning point of entrepreneurial cognition research. Some empirical studies have been carried out gradually, and the previous theoretical hypotheses have been tested. Since the cognitive perspective provides a rich theoretical basis and more perfect research methods for entrepreneurship research, the research on entrepreneurship cognition has become mature and gradually developed into an independent research field (Sassetti et al., 2018). The current research on entrepreneurial cognition mainly revolves around two theoretical issues: On the one hand, because individuals cannot be entirely rational, coupled with the high degree of uncertainty and resource constraints of the entrepreneurial environment, many aspects of entrepreneurial cognition are dominated by various cognitive biases, such as counterfactual thinking (Hao et al., 2018) and overconfidence (Lee et al., 2015). Therefore, research on entrepreneurial cognition focuses on various cognitive biases on entrepreneurial behavior and decision-making and has achieved rich results.

On the other hand, how does the entrepreneur’s unique cognition model affect entrepreneurial results (excellent results)? For example, researchers have shown that entrepreneurial cognition affects opportunity recognition (Kemmerer et al., 2012; Renko et al., 2012; Dew et al., 2015). Higher levels of knowledge (education) usually seem to help identify opportunities, but different types of knowledge trigger the different recognition of opportunities (Shepherd and Patzelt, 2018). In addition, research on entrepreneurial cognition also pays attention to the differences between entrepreneurs and managers in risk perception and self-efficacy to highlight the uniqueness of entrepreneurs’ cognition. Therefore, the cognitive perspective can effectively distinguish entrepreneurs from other individuals, which is significant for explaining the definition of entrepreneurs, entrepreneurial behavior, and entrepreneurial success.

Entrepreneurial cognition theory holds that the critical reason for entrepreneurs’ entrepreneurial success is that they have a more reasonable entrepreneurial cognition. Entrepreneurs can use their entrepreneurial cognition to integrate scattered information to create new products and services and use their entrepreneurial cognition to integrate necessary resources and start new businesses. Successful entrepreneurs have cognitive resources on identifying entrepreneurial opportunities, obtaining entrepreneurial resources, exploring entrepreneurial models, and summarizing these cognition resources into three dimensions: arrangement scripts, willing scripts, and ability scripts, entrepreneurial success results from the simultaneous action of the three scripts (Mitchell et al., 2000). Following academia has carried out related research on entrepreneurial cognition from multiple levels. Lim et al. (2017) took eight countries as examples to study the impact of the social system and environmental factors on entrepreneurial cognition. They pointed out that entrepreneurial social capital will affect various types of entrepreneurial cognition. Cognitive bias affects entrepreneurial performance; cultural values will affect entrepreneurial cognitive development (Busenitz and Lau, 1996; Mitchell et al., 2000). Although these studies all promote the development of entrepreneurial cognition theory, they mainly study entrepreneurial cognition from the macro level. Even at the individual level, they also focus on the external social factors affecting individual behavior, while the research on its internal factors is insufficient.

### Positive Emotions

Emotions are a complex phenomenon composed of multiple components, such as sensory states, action systems, and the process of organizing cognition and behavior. The most important parts of emotion are emotional valence, arousal, and motivational direction (Lacey et al., 2021). Positive emotions are good and pleasant feeling when the individual completes the target task and is affirmed by the outside world. This kind of pleasure not only can meet individual needs and then help individuals to carry out activities smoothly (Cabanac, 2003; Carver, 2003) but enhance the initiative and vitality of individual behavior (Davidson and Irwin, 1999). The broaden-and-build theory of positive emotions believes that positive emotions expand the cognition range of individuals, increase the number of cognition resources that can be used for connection, distract attention to more complex situations, and increase related resources that can be used to solve problems. It also restructures the action command system to encourage individuals to pursue novel and creative thoughts and action paths, thereby generating positive “emotion-cognition-behavior” spiraling forces (Fredrickson, 2001). The ideas and behaviors triggered by positive emotions are more creative and flexible, whether spontaneous or induced positive emotions (Davis, 2009; Talarico et al., 2009; Johnson et al., 2010). Compared with negative and neutral emotions, positive emotions significantly impact individual creativity (Baas et al., 2008). Positive emotions are conducive to promoting team members’ cognition development and spontaneous learning. They are more willing to put forward new ideas based on others’ ideas and encourage task solving and team innovation (Rhee, 2006; Yuan and Wu, 2020). In summary, positive emotions can expand individual cognition and stimulate individual innovative thinking. In a positive emotional state, individuals are more likely to develop new ideas, try new methods, and solve problems or achieve more flexibly.

## Research Hypotheses

### Positive Emotions and Entrepreneurial Cognition

Entrepreneurship cognitions are composed of three scripts: arrangement scripts, willing scripts, and ability scripts. Entrepreneur’s entrepreneurial activities are started under the combined action of these three types of scripts (Mitchell et al., 2000). The so-called scripts are the knowledge structures that people use to connect cognitions and behaviors. People’s memory and experience are stored in the brain in the form of scripts. When an individual is in a particular situation or is stimulated by a specific external environment, the brain will extract the knowledge structure related to the current climate and take action accordingly. Arrangement of entrepreneurship is a preparatory activity for various resources, capabilities, relationships, and other conditions required for entrepreneurial activities. The arrangement scripts are the knowledge structures of these activities to realize creative protection, entrepreneurial network acquisition, entrepreneurial resource acquisition, and entrepreneurial expert-level skills development in specific fields. The willing scripts refer to the knowledge structures about risks and rewards entrepreneurs possess in the entrepreneurial process. People with these knowledge structures seek new areas and things with an open mind and a focused spirit, implement action steps, and assume entrepreneurial risks. As the creators of new enterprises, entrepreneurs have more robust willing scripts than ordinary people, and the willing scripts explain the intrinsic motivation of entrepreneurs. The ability scripts refer to the necessary abilities, knowledge, and skills for entrepreneurship, including risk diagnosis scripts, contextual knowledge scripts, and ability-opportunity matching scripts. The ability scripts are the performance of entrepreneurs’ ability and directly affect entrepreneurial performance.

Modern positive emotions theory provides a new perspective for this article to understand entrepreneurial cognitive thinking. Positive emotions and rewards can have various effects on cognitive control (Chiew, 2021). Under the condition of low-level approach motivation, positive emotions can increase flexibility and expand attention, while high-level approach motivation can increase goal maintenance and little attention (Paul et al., 2021). Positive emotions influence the content of cognitive processing and influence the cognition processing process (Bower, 1991). Based on this, this article proposes that positive emotions impact entrepreneurial cognition; in other words, positive emotions positively affect the arrangement scripts, willing scripts, and ability scripts. The following hypotheses are proposed:

*H1*: Positive emotions have a positive impact on arrangement scripts of entrepreneurial cognition.

*H2*: Positive emotions have a positive impact on willing scripts of entrepreneurial cognition.

*H3*: Positive emotions have a positive impact on ability scripts of entrepreneurial cognition.

### Positive Emotions and Entrepreneurial Intention

The entrepreneur’s emotion refers to an emotion held by an individual or a team in entrepreneurship, which includes both personal mood and personal feelings. This emotion may exist before the entrepreneurial process and may be accompanied by the entrepreneurial process (Cardon et al., 2012). The time relationship between the conscious intention of the action and the action itself will change with the change of the emotional state, and positive emotions may enhance the intentional consciousness (Davide et al., 2015). As an individual’s possibility and psychological commitment to creating a new enterprise, entrepreneurship intention is a possibility of individual self-employment. Entrepreneur’s positive emotion is the key factor that encourages individuals to produce entrepreneurial activities. Entrepreneur’s positive emotions enhance the entrepreneurial desire of potential entrepreneurs, and the improved entrepreneurial intention drives entrepreneurial behavior. Because this is a conscious experience of the individual, it can help the individual better engage in activities. In the entrepreneurial process, positive emotions may significantly affect the individual entrepreneurial intention, in turn, affect their behavior and state, which helps entrepreneurs to actively cope with high uncertainty and high risk of the business environment, thus form risk-taking behavior, and take an upward emotional state of mind, positive commitment for a long time. Based on this, this article believes that positive emotions have a positive impact on entrepreneurial intention. Put forward the following hypothesis:

*H4*: Positive emotions have a positive impact on entrepreneurial intention.

### Entrepreneurial Cognition and Entrepreneurial Intention

There is an excellent relationship between entrepreneurial cognition and the formation of entrepreneurial intention. Ajzen (1991) held the view that exogenous variables indirectly affect entrepreneurial motivation and entrepreneurial behavior. Shaver and Scott (1992) deemed that if the stimulus-response model cannot fully explain entrepreneurial motivation and entrepreneurial behavior, the relationship between entrepreneurial cognition theory and entrepreneurial motivation can be verified from the perspective of perceptual cognition. Krueger (2007) considered that entrepreneurial action comes from willingness, willingness comes from the attitude, and the attitude comes from the deep cognitive structure. Entrepreneurship is to find the factors behind entrepreneurial cognition, entrepreneurial intention, and entrepreneurial action of individuals. Entrepreneurs will have the perception of entrepreneurial opportunities and the willingness to pursue opportunities before taking entrepreneurial actions. According to the entrepreneurial cognitive schema, entrepreneurial self-efficacy, and the development of entrepreneurial intention, an analysis framework of entrepreneurial cognition is proposed, and the causes of entrepreneurial intention are backward explored by tracing back to the root.

This article uses a Entrepreneurial cognitive concept of Mitchell et al. (2000) to explain the relationship between entrepreneurial cognition and entrepreneurial intention. His entrepreneurial cognition concepts include arrangement scripts, willing scripts, and ability scripts. The arrangement scripts for entrepreneurship include all the necessary resources needed to create a new business. Entrepreneurs will try their best to make use of the entrepreneurial resources around them in the entrepreneurial process. People with more knowledge structure of arrangement scripts will make better use of social resources to make sufficient entrepreneurial preparations and always pay attention to the changes in the entrepreneurial environment to ensure that their decisions can be recognized by others and improve the accuracy of decisions. The willing scripts can make entrepreneurs feel the less entrepreneurial risk and promote entrepreneurial decision-making. The higher the knowledge structures of willing scripts, the fewer entrepreneurial risks that entrepreneurs perceive. When making decisions, they only pay attention to the primary information of entrepreneurship, and less consider other irrelevant information, the faster the entrepreneurial decision-making speed. In terms of ability scripts, the entrepreneurship ability scripts have an important influence on entrepreneurial decision-making programs. Entrepreneurs with a rich knowledge structure of ability scripts can more comprehensively collect and process various decision-making information. They thoroughly and comprehensively analyze the entrepreneurial environment, select the most suitable decision-making plan to improve the accuracy of entrepreneurial decision-making, and be recognized by other entrepreneurial partners. Accordingly, this study puts forward the following hypotheses:

*H5*: The arrangement scripts of entrepreneurial cognition have a positive impact on entrepreneurial intention.

*H6*: The willing scripts of entrepreneurial cognition have a positive impact on entrepreneurial intention.

*H7*: The ability scripts of entrepreneurial cognition have a positive impact on entrepreneurial intention.

### The Mediating Effect of Entrepreneurial Cognition

Cognition can control the emotional response, which in turn can promote the development of decision-making behavior. Emotions influence decision-making by influencing individual cognition and the use of metacognitive strategies (Martin and Delgado, 2011). Emotions provide entrepreneurs with specific information for judging the value of entrepreneurial opportunities, thereby dictating their attitudes and thinking styles and influencing entrepreneurial behavior (Chung et al., 2013).

Cognitive Appraisal Theory of Emotion believes that individuals will automatically respond emotionally to this stimulus when facing pressure, assess the threats or challenges they will face (trigger cognition), and then affect the subsequent willingness and behavioral responses (Folkman, 1984). Entrepreneurial cognition ability will be reflected through the cognition model, cognition structure, and cognition content. Therefore, the overconfidence and control illusion in the cognitive bias of entrepreneurs can promote their entrepreneurial intention and continuous entrepreneurial behavior (Simon et al., 2000); more entrepreneurial cognition will enable individuals to understand the complexity and uncertainty of the entrepreneurial environment quickly and then decide whether to carry out the preparatory work for entrepreneurship (Mitchell et al., 2002). According to the Affect Priming Theory, emotions can affect the judgment process of individuals by triggering people’s information retrieval, coding, and selection of practical information and have an impact on personal cognition (Podoynitsyna et al., 2012). Specifically, individuals with positive emotions are more likely to favor optimistic assessments and judgments and then seek risks, and individuals with negative emotions are more likely to favor pessimistic evaluations and judgments, thereby avoiding risks. Naturally, entrepreneurial emotions will affect the individual’s entrepreneurial intention through the impact on entrepreneurial cognition. Mitchell et al. (2002) also confirmed the mediating role of entrepreneurial cognition in the entrepreneurial atmosphere. In summary, the following hypotheses are proposed:

*H8*: The arrangement scripts of entrepreneurial cognition partly mediate between positive emotions and entrepreneurial intention.

*H9*: The willing scripts of entrepreneurial cognition partly mediate between positive emotions and entrepreneurial intention.

*H10*: The ability scripts of entrepreneurial cognition partly mediate between positive emotions and entrepreneurial intention.

**Figure 1:**
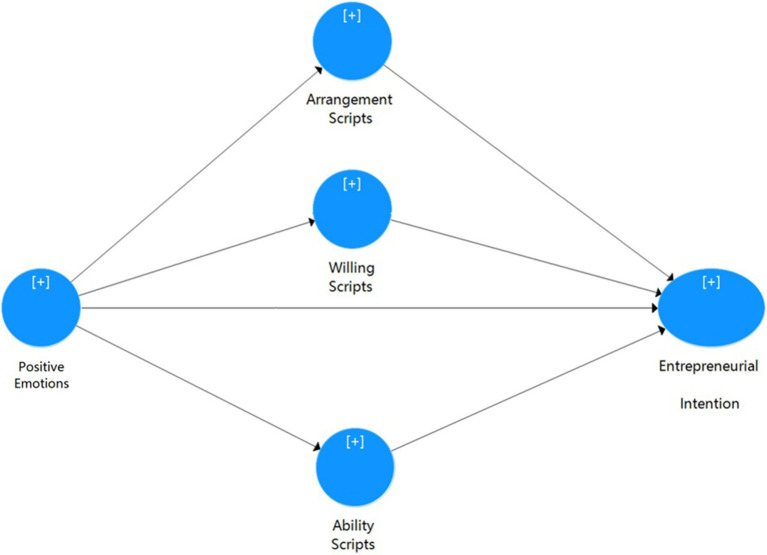
Research model.

This article refers to two classic entrepreneurial intention models: Entrepreneurial Event Model and Theory of Planned Behavior. We combined the two theoretical models, simplified them, and retained the basic framework. Taking positive emotions and entrepreneurial cognition as factors that affect entrepreneurial intentions and at the same time using entrepreneurial cognition as a mediating variable of positive emotions and entrepreneurial intentions, the model of this study is established. Figure 1 showed the research model.

## Methodology

In order to verify the foregoing hypotheses, we used the method of questionnaire, which includes three processes: selecting samples and sample demographic data, measures, and data analysis.

### Sample

The samples selected in this study need to meet the following three conditions: (1) Potential entrepreneurs, who have not started their business in a real business environment, but are likely to do so in the future. (2) Who have a certain experience in innovation and entrepreneurship, such as participating in entrepreneurship courses and entrepreneurship training projects or entrepreneurial competitions. (3) Who have a certain experience of entrepreneurial emotion or entrepreneurial cognition in the practice of innovation and entrepreneurship.

The data were collected from students from 21 colleges and universities participating in the “3chuang” Challenge in Guangdong Province, such as Sun Yat-sen University, South China Normal University, Guangdong University of Technology, Shenzhen University, UESTC, and Zhongshan Institute. These students participated in at least one “3chuang” Challenge. The “3chuang” Challenge runs through the entire academic year of the university. From September to July of the second year, it goes through three stages: school competition-provincial competition-national competition. Since the survey participants participated in the “3chuang” Challenge, they have experience not only in innovation and entrepreneurship, but also in emotions and cognition in the practice of innovation and entrepreneurship, which has established a good foundation for this research. Questions about gender, grade, and major were set up in the questionnaire regarding demographic data. A total of 297 students were given questionnaires. Nine questionnaires that took less than 60s to fill in and incompletely filled in were deleted, and 288 valid questionnaires were returned. The general information of the interviewed students is as follows (Table 1). It can be seen that there are more girls than boys, which may have a lot to do with the students’ majors and the girls’ enthusiasm for the survey. Participating in the “3chuang” Challenge is mainly for freshmen and sophomores. This is because on the one hand, lower-grade students are actively participating in the competition, and on the other hand, lower-grade students are less stressed in their studies, advancement, and work. This is also the objective reason why this study investigates entrepreneurial intention rather than entrepreneurial decisions and behaviors. The survey subjects are mainly students majoring in management (especially e-commerce), which is primarily determined by the nature of competition.

**Table 1:** Demographic profile of respondents.

Demographic profile		Frequency	Percentage
Gender	Male	94	32.64%
	Female	194	67.36%
Grade	Freshman	71	24.65%
	Sophomore	187	64.93%
	Junior	23	7.99%
	Senior	7	2.43%
Major	Electronic commerce	89	30.90%
	Financial management	24	8.33%
	Finance	44	15.28
	International trade	51	17.71
	Management	16	5.56%
	Human resource	7	2.43%
	Law	5	1.74%
	Logistics management	3	1.04%
	Major related to Computer Science	9	3.13%
	Major related to English	6	2.08%
	Automation	4	1.39%
	Major related to Journalism and Media	4	1.39%
	Others (Food, marine resources, administration, nuclear industry, environment, educational technology, tourism, etc.)	26	9.03%

### Measures

As shown in Table 2, according to the hypotheses, this questionnaire would set up a measurement scale for positive emotions, three scripts of entrepreneurial cognition (arrangement scripts, willing scripts, and ability scripts), and entrepreneurial intention. The measurement scale was carefully adapted from the sources listed in Table 2 and the characteristics of the survey subjects. Positive emotions extracted four items from the scale developed by Watson et al. (1988), such as “When participating in “3chuang” and I’m interested.” According to the definition of Mitchell et al. (2000), entrepreneurial cognition is divided into arrangement scripts, willing scripts, and ability scripts. In this study, referring to the scale developed by Mitchell et al. (2000) and combining with the characteristics of the subject of the survey, four arrangement scripts were extracted, such as “My new venture is/will be protected from competition by patent, secret technology or knowledge,” five willing scripts were extracted, such as “Do you want things open to the possibilities?,” and four ability scripts were extracted, such as “When I see a business opportunity I decide to invest based upon how closely it fits my ‘success scenario’.” Entrepreneurial intention adopted the scale developed by Liñán et al. (2011), such as “I am ready to make anything to be an entrepreneur.” The questionnaire used the Likert five-point scale to measure these indicators, ranging from “1-strongly disagree” to “5-strongly agree.”

**Table 2:** Indicators for constructs.

Constructs	Indicators	Sources
Positive emotions	PE1- When participating in “3chuang,” I am interested.	Watson et al., 1988
	PE2- When participating in “3chuang,” I am enthusiastic.	
	PE3- When participating in “3chuang,” I am proud.	
	PE4- When participating in “3chuang,” I am inspired.	
Arrangement scripts	ArS1- My new venture is/will be protected from competition by patent, secret technology, or knowledge.	Mitchell et al., 2000
	ArS2- I can often see opportunities for my plans to fit with those of other people.	
	ArS3- I could raise resources for a venture if I did not have enough.	
	ArS4- I am very good at a specialty that is in high demand.	
Willing scripts	WS1-Do you want things open to possibilities?	
	WS2-I am more comfortable in new situations.	
	WS3-I do not mind being committed to meeting a regular payroll if I can have a chance at tremendous financial success.	
	WS4-I am looking for a place to invest my resources.	
	WS5-When investing in a new venture, I think it is worse to wait too long and miss a great opportunity.	
Ability scripts	AbS1-When I see a business opportunity, I decide to invest based upon how closely it fits my “success scenario.”	
	AbS2-I often see ways in which a new combination of people, materials, or products can be of value.	
	AbS3-When confronted with a new venture problem, I can vividly recall the details of similar situations I know about.	
	AbS4-When someone describes a problem with a new business, I can quickly recognize key features of the problem and suggest alternatives from examples I can cite.	
Entrepreneurial intention	EI1-I am ready to make anything to be an entrepreneur.	Liñán et al., 2011
	EI2-My professional goal is to become an entrepreneur.	
	EI3-I will make every effort to start and run my own firm.	
	EI4-I am determined to create a firm in the future.	
	EI5-I have very seriously thought in starting a firm.	
	EI64-I have got the firm intention to start a firm someday.	

### Data Analysis

This model verification uses the Smart PLS3 tool. PLS-SEM is a structural equation model based on variance. Compared with the first-generation structural equation model based on covariance, PLS-SEM has more robust statistical capabilities (Hair et al., 2012). In addition, PLS-SEM can process data with non-normal distribution (Hair et al., 2014b; Sarstedt et al., 2014). According to the suggestions of Lee et al. (2016), a single-sample Kolmogorov-Smirnov normality test was used to verify the data distribution, and the results showed that the data had non-normal distribution. Therefore, PLS-SEM is considered suitable for this study. A two-step method is strictly followed in the PLS-SEM analysis, namely, checking the measurement and structural models (Hew et al., 2017). In addition, the common method bias, predictive power of the model, and mediation are evaluated and reported (Lowry and Gaskin, 2014).

## Results

### Common Method Bias Analysis

When the responses of exogenous and endogenous variables are from the same respondent, common method variance is a potential problem (Chang et al., 2010). So, this study includes some measures to control this bias (Hew et al., 2016). First and foremost, use simple language to ensure that the items in the questionnaire are concise enough during the questionnaire design stage. Second, in the questionnaire management stage, the respondents were guaranteed anonymity and informed that there is no right or wrong answer to each item. In addition, to ensure that the common method bias is not a serious problem in this study, we used PLS to perform the common method bias test according to the recommendations of Liang et al. (2007). Table 3 shows that most of the method factor loadings are negative and insignificant, and the average substantive variance of the indicators is 0.791, the average method variance is 0.018, and the ratio of the two is 43:1. Therefore, the problem of common method bias in this study should not be severe.

**Table 3:** An alternative test for common method bias.

Constructs	Indicators	Substantive factor loading (*Ra*)	Substantive variance (*Ra*^2^)	Method factor loading (*Rb*)	Method variance (*Rb*^2^)
Positive emotions	PE1	0.902^***^	0.814	-0.165^NS^	0.027
	PE2	0.917^***^	0.841	-0.104^NS^	0.011
	PE3	0.899^***^	0.808	0.045^NS^	0.002
	PE4	0.852^***^	0.726	0.042^NS^	0.002
Arrangement scripts	ArS1	0.918^***^	0.843	0.108^*^	0.012
	ArS2	0.890^***^	0.792	0.087^NS^	0.008
	ArS3	0.886^***^	0.785	-0.109^NS^	0.012
	ArS4	0.873^***^	0.762	-0.145^NS^	0.021
Willing scripts	WS1	0.881^***^	0.776	0.013^NS^	0.000
	WS2	0.867^***^	0.752	-0.054^NS^	0.003
	WS3	0.887^***^	0.787	-0.089^NS^	0.008
	WS4	0.921^***^	0.848	-0.071^NS^	0.005
	WS5	0.902^***^	0.814	0.217^(*)^	0.047
Ability scripts	AbS1	0.887^***^	0.787	-0.254^NS^	0.065
	AbS2	0.922^***^	0.850	0.153^NS^	0.023
	AbS3	0.911^***^	0.830	-0.158^NS^	0.025
	AbS4	0.893^***^	0.797	0.259^NS^	0.067
Entrepreneurial intention	EI1	0.853^***^	0.728	0.056^NS^	0.003
	EI2	0.863^***^	0.745	0.228^*^	0.052
	EI3	0.893^***^	0.797	-0.079^NS^	0.006
	EI4	0.888^***^	0.789	0.118^NS^	0.014
	EI5	0.881^***^	0.776	-0.037^NS^	0.001
	EI6	0.864^***^	0.746	-0.068^NS^	0.005
Average			0.791		0.018
Ratio		43.485			

NS, insignificant.

*** *p*<0.001;

* *p*<0.05;

(*) *p*<0.10.

### Inspecting the Measurement Model

In the process of inspecting the measurement model, the reliability and validity requirements must be met simultaneously (Hair et al., 2014b). Reliability can be established using composite reliability, which has to be more than 0.70 (Hair et al., 2012). As shown in Table 4, reliability is fulfilled by all constructs.

**Table 4:** Reliability and convergent validity.

Constructs	Mean	*SD*	Composite reliability	Average variance extracted
Positive emotions	3.653	1.16075	0.940	0.797
Arrangement scripts	3.345	1.079	0.940	0.795
Willing scripts	3.743	1.044	0.951	0.795
Ability scripts	3.455	1.009	0.947	0.816
Entrepreneurial intention	3.176	1.231	0.951	0.764

Following Sarstedt et al. (2014), two types of validity need to be established: convergent and discriminant validity. From their description, convergent validity can be confirmed if the indicator loadings are higher than 0.70 and the average variance extracted for each construct is more significant than 0.50. Tables 4 and 5 both show that the convergence validity is fulfilled. In contrast, the cross-loading of the criteria and indicators (Sarstedt et al., 2014) by Fornell and Larcker (1981) can detect discriminant validity. In the standard of Fornell and Larcker (1981), the square root of the average extracted variation of each construct must be greater than the inter-construct correlations. Table 6 shows that this criterion is satisfied. In addition, according to Table 5, the load value of the reflection index of each construct is higher than the load value of the other construct, so the discriminant validity is confirmed again. Henseler et al. (2016) believe that the discriminant validity needs to be verified by another criterion: the heterogeneity-elemental substance ratio must be significantly less than 1. Table 7 shows that this criterion has also been met.

**Table 5:** Factorloading and cross-loading.

	Positive emotions	Arrangement scripts	Willing scripts	Ability scripts	Entrepreneurial intention
PE1	**0.899**	0.647	0.664	0.641	0.561
PE2	**0.916**	0.649	0.704	0.659	0.514
PE3	**0.897**	0.650	0.72	0.642	0.486
PE4	**0.858**	0.727	0.697	0.677	0.580
ArS1	0.643	**0.914**	0.627	0.641	0.604
ArS2	0.724	**0.891**	0.742	0.730	0.604
ArS3	0.622	**0.882**	0.619	0.656	0.604
ArS4	0.681	**0.879**	0.685	0.737	0.714
WS1	0.697	0.639	**0.879**	0.673	0.501
WS2	0.711	0.733	**0.868**	0.670	0.551
WS3	0.680	0.662	**0.888**	0.680	0.624
WS4	0.737	0.695	**0.922**	0.709	0.640
WS5	0.652	0.617	**0.900**	0.671	0.571
AbS1	0.655	0.652	0.663	**0.886**	0.667
AbS2	0.678	0.706	0.702	**0.923**	0.753
AbS3	0.666	0.735	0.706	**0.912**	0.708
AbS4	0.655	0.715	0.688	**0.893**	0.684
EI1	0.572	0.661	0.627	0.739	**0.859**
EI2	0.468	0.638	0.505	0.658	**0.863**
EI3	0.591	0.648	0.638	0.700	**0.895**
EI4	0.518	0.616	0.567	0.681	**0.887**
EI5	0.521	0.591	0.541	0.675	**0.879**
EI6	0.470	0.561	0.515	0.618	**0.860**

Bold values mean factorloading, other values are cross-loading. PE, positive emotions; ArS, arrangement scripts; WS, willing scripts; AbS, ability scripts; and EI, entrepreneurial intention.

**Table 6:** Fornell and Larcker criterion.

	AVE	Positive emotions	Arrangement scripts	Willing scripts	Ability scripts	Entrepreneurial intention
Positive emotions	0.797	***0.893***				
Arrangement scripts	0.795	0.75	***0.892***			
Willing scripts	0.816	0.781	0.752	***0.892***		
Ability scripts	0.795	0.734	0.777	0.764	***0.903***	
Entrepreneurial intention	0.764	0.601	0.711	0.65	0.779	***0.874***

The square root of the average variance extracted for each construct is denoted in bold and italic, while the inter-construct correlations are shown off-diagonally.

**Table 7:** The Heterotrait-Monotrait ratio of correlations criterion.

	Positive emotions	Arrangement scripts	Willing scripts	Ability scripts	Entrepreneurial intention
Positive emotions					
Arrangement scripts	0.817				
Willing scripts	0.843	0.809			
Ability scripts	0.798	0.843	0.821		
Entrepreneurial intention	0.645	0.762	0.689	0.833	-

### Examining the Structural Model

**Figure 2:**
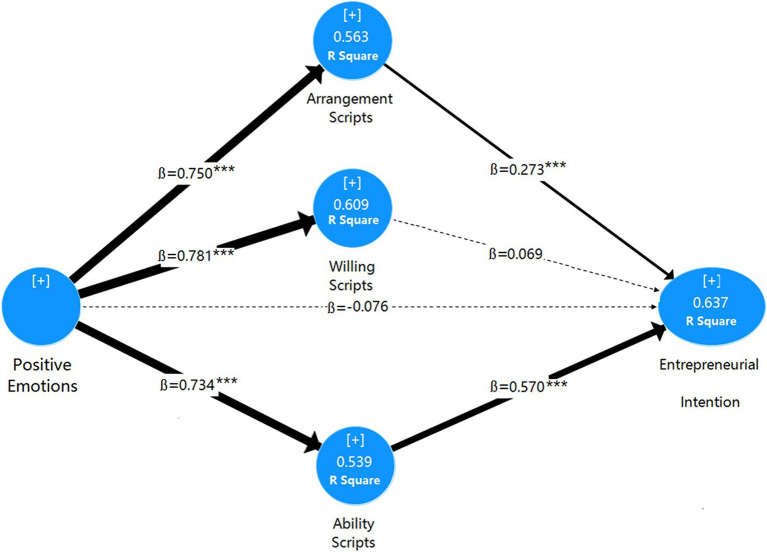
The outcome of the structural model examination. ***p < 0.001; →, significant path; ⇢, insignificant path.

Given the sound measurement model, the next step was to examine the structural model to verify the theoretically established paths statistically and confirm the developed hypotheses. A bootstrapping procedure with 5,000 sub-samples was performed through SmartPLS 3 with the “no sign change” option, which delivers the most conservative outcomes (Hair et al., 2014a). The outcome of the examination is listed in Table 8 and displayed in Figure 2 for better illustration. At the same time, we evaluated the size of direct and indirect effects among the five constructs, and the results are shown in Table 9.

**Table 8:** Outcome of structural model examination.

Hypotheses	Paths	Path coefficients	T Statistics	*p*	CI bias corrected	Remarks
H1	Positive emotions->Arrangement scripts^***^	0.750	26.066	0.000	[0.687, 0.801]	Supported
H2	Positive emotions->Willing scripts^***^	0.781	28.807	0.000	[0.720, 0.829]	Supported
H3	Positive emotions->Ability scripts^***^	0.734	23.900	0.000	[0.668, 0.789]	Supported
H4	Positive emotions->Entrepreneurial intention^NS^	−0.076	0.980	0.327	[−0.228, 0.073]	Unsupported
H5	Arrangement scripts->Entrepreneurial intention^***^	0.273	3.710	0.000	[0.125, 0.417]	Supported
H6	Willing scripts->Entrepreneurial intention^NS^	0.069	0.808	0.419	[−0.094, 0.235]	Unsupported
H7	Ability scripts->Entrepreneurial intention^***^	0.570	8.204	0.000	[0.432, 0.706]	Supported

NS, insignificant.

*** *p*<0.001.

**Table 9:** Multiple mediation analysis.

Hypotheses and paths	Specific indirect	Direct effect	Total effect	Types of mediation	Remarks
H8: Positive emotions->Arrangement Scripts->Entrepreneurial intention	0.205^***^	−0.076^NS^	0.601^***^	Full mediation	Supported
H9: Positive emotions->Willing Scripts->Entrepreneurial intention	0.054^NS^	−0.076^NS^	0.601^***^	No effect non-mediation	Unsupported
H10: Positive emotions->Ability Scripts->Entrepreneurial intention	0.418^***^	−0.076^NS^	0.601^***^	Full mediation	Supported

NS, insignificant.

*** *p*<0.001.

Positive emotions as an irrational factor in the model influence the three scripts of entrepreneurial cognition as rational factors: Positive emotions positively significantly influence the arrangement scripts (*ß*=0.750, *p*<0.001), positive emotions positively significantly influence willing scripts (*ß*=0.781, *p*<0.001), positive emotions positively significantly influence ability scripts (*ß*=0.734, *p*<0.001), but positive emotions do not influences entrepreneurial intention (*ß*=−0.076, *p*=0.327>0.1). In addition, the arrangement scripts positively significantly influence the entrepreneurial intention (*ß*=0.273, *p*<0.001), and the ability scripts positively significantly influence the entrepreneurial intention (*ß*=0.570, *p*<0.001), but the intention scripts do not influence the entrepreneurial intention (*ß*=0.069, *p*=0.419>0.1). Therefore, H1, H2, H3, H5, and H7 are supported, and H4 and H6 are unsupported. Overall, the multiple mediation model can account for 63.7% of the variance in entrepreneurial intention (*R*^2^=0.637).

### Predictive Relevance and Effect Size

The *R*^2^ value of the endogenous latent variable in the structural model is 0.75, 0.50, or 0.25, which is expressed as substantial, medium, or weak (Hair et al., 2011). Surveyed data executed 5,000 bootstrapping procedure model operations using SmartPLS3 to evaluate the significance of the path coefficient. The critical *t* values for the two-tailed test are 1.65 (significance level=10%), 1.96 (significance level=5%), and 2.58 (significance level=1%; Hair et al., 2011). It can be seen from Table 10 that *R*^2^ values are all greater than 0.50, and the path coefficients are significant except for “Positive emotions->Entrepreneurial Intention” and “Willing Scripts->Entrepreneurial Intention,” which are non-significant, indicating that except for these two insignificant paths, the predictive accuracy of the model is between substantial and medium.

**Table 10:** Predictive relevance and effect size.

Endogenous variables	*R* ^2^	*Q*^2^ (=1-SSE/SSO)	Exogenous variables	Effect size *f*^2^
Arrangement scripts	0.563	0.442	Positive emotions	1.289
Willing scripts	0.609	0.480	Positive emotions	1.56
Ability scripts	0.539	0.436	Positive emotions	1.171
Entrepreneurial intention	0.637	0.479	Positive emotions Arrangement scripts Willing scripts Ability scripts	0.005
				0.063
				0.004
				0.277

In addition to using the coefficient of determination (*R*^2^) to assess the predictive accuracy of a model, Hair et al. (2014a,b) suggested using cross-validated redundancy (*Q*^2^) to ascertain the predictive relevance of the model. If the *Q*^2^ value of the endogenous variable is greater than zero, the model can be considered to have predictive relevance for it (Hair et al., 2017). It can be seen from Table 10 that the multi-mediation model has predictive relevance to the arrangement scripts, willing scripts, ability scripts, and entrepreneurial intention.

In addition, the effect size *f*^2^ for each of the exogenous variables is calculated in Table 10. According to Hair et al. (2014a,b), the effect size *f*^2^ represents the exogenous variable’s contribution to the *R*^2^ value of the endogenous variable, and the values of 0.02, 0.15, and 0.35 represent small, medium, and large effects. Thus, it can be seen from Table 10 that positive emotions have large effects on the arrangement scripts, willing scripts, and ability scripts of entrepreneurial cognition. In contrast, the effects of arrangement scripts and ability scripts on entrepreneurial intention are small and medium, respectively.

### Analyzing the Multiple Mediating Effects

This study uses the methods proposed by Zhao et al. (2010) and Hair et al. (2017) to analyze multiple mediation effects. According to their description, there are two types of non-mediation and three types of mediation. If the direct and indirect effects of the mediation between the independent and dependent variables are not significant, then the path has no effect non-mediation. However, if only the direct effect is significant, the path is only direct non-mediation; if only the indirect effect is significant, researchers need to evaluate further the significance of the direct effect to further distinguish between complementary partial mediation, competitive partial mediation, and full mediation. When the direct effect is significant and consistent with the direction of the indirect effect, a complementary partial mediation occurs; when the direct effect is significant, but the direction of the indirect effect is opposite, a competitive partial mediation occurs. Finally, full mediation only exists when the direct effect is not significant. In addition, researchers should check whether the total effect for each path of establishing mediation is significant. It can be seen from Table 9 that the arrangement scripts and the ability scripts play the role of full mediators between positive emotions and entrepreneurial intention, while the mediation effect of willing scripts detection is not significant, and there is no effect non-mediation. Therefore, H8 and H10 are supported, while H9 is unsupported.

## Discussion

This research surveyed the group of college students participating in the “3chuang” competition. This group has the foundation of learning knowledge and entrepreneurial cognition and has practical embryo experience in innovation and entrepreneurship through the competition, but there is still a long distance from real entrepreneurship. This research establishes two influencing factors that affect entrepreneurial intention: positive emotions and entrepreneurial cognition – one as an irrational factor and the other as a rational factor. By establishing a structural equation model, it is initially assumed that irrational positive emotions and rational entrepreneurial cognition affect entrepreneurial intention together and that rational entrepreneurial cognition has a mediating effect between irrational positive emotions and entrepreneurial intention. Through the empirical analysis calculated by PLS-SEM, most of the hypotheses have been verified, but positive emotions do not affect entrepreneurial intention. The arrangement and ability scripts in entrepreneurial cognition fully mediate between positive emotions and entrepreneurial intention, while willing scripts have no mediating effect, as shown in Table 11. Several issues need further discussion.

**Table 11:** The Results of hypotheses.

Hypotheses	Remarks
H1: Positive emotions have a positive impact on arrangement scripts of entrepreneurial cognition	Supported
H2: Positive emotions have a positive impact on willing scripts of entrepreneurial cognition	Supported
H3: Positive emotions have a positive impact on ability scripts of entrepreneurial cognition	Supported
H4: Positive emotions have a positive impact on entrepreneurial intention	Unsupported
H5: The arrangement scripts of entrepreneurial cognition have a positive impact on entrepreneurial intention	Supported
H6: The willing scripts of entrepreneurial cognition have a positive impact on entrepreneurial intention	Unsupported
H7: The ability scripts of entrepreneurial cognition have a positive impact on entrepreneurial intention	Supported
H8: The arrangement scripts of entrepreneurial cognition partly mediate between positive emotions and entrepreneurial intention	Supported
H9: The willing scripts of entrepreneurial cognition partly mediate between positive emotions and entrepreneurial intention	Unsupported
H10: The ability scripts of entrepreneurial cognition partly mediate between positive emotions and entrepreneurial intention	Supported

### The Effect of Positive Emotions on Entrepreneurial Cognition

The positive emotions examined in this study are those of college students participating in innovation and entrepreneurship competitions. There is a distance between innovation and entrepreneurship competitions and real entrepreneurship, and there is a large gap in the resources, time, and energy needed. From the survey results, positive emotions positively and significantly affect entrepreneurial cognition. First, positive entrepreneurial emotions help entrepreneurs evaluate the entrepreneurial environment with optimism and expand their cognition scope to evaluate entrepreneurial opportunity information on time. Because positive emotions can expand the scope of individuals’ search for things and information to carry out precise addition of information (Rhee, 2006). Second, if the information content is consistent with the positive emotions, entrepreneurs can enhance their ability to encode and remember relevant information. Third, positive entrepreneurial emotions can prompt entrepreneurs to break the original mental model when dealing with entrepreneurial activities, improve creativity, and achieve success. Finally, individuals with positive emotions are more likely to grasp entrepreneurial opportunities, integrate entrepreneurial resources, and make proactive decisions with confidence.

### The Effect of Entrepreneurial Cognition on Entrepreneurial Intention

From the scale of the willing scripts, it is the knowledge structure between entrepreneurial risk and entrepreneurial return possessed by entrepreneurs. The empirical research concludes that the willing scripts do not influence the entrepreneurial intention. Does this mean that the group of college students be fond of eating and averse to work? From the perspective of mediating effect, arrangement scripts and ability scripts have a completely mediating effect on positive emotions and entrepreneurial intention, while willing scripts have no mediating effect. Arrangement scripts are the knowledge structure entrepreneurs use to prepare for the various resources, abilities, and relationships needed to start entrepreneurial activities. The ability scripts are the knowledge structure of entrepreneurs’ abilities, knowledge, and skills when starting a business. In other words, entrepreneurs only have the knowledge structure of arrangement scripts and ability scripts, positive emotions to play a role. According to the entrepreneurial process model proposed by Timmons (2008), the entrepreneurial process is the result of matching and balancing the three elements of business opportunities, entrepreneurs, and resources. In the ambiguous and uncertain dynamic entrepreneurial environment, the founder or work team must have the ability to creatively capture business opportunities, integrate resources, build strategies, and solve problems, work diligently, and be rich in sacrifices. Does this mean that the survey respondents must first have opportunities and resources to start a business and are unwilling to contribute and take risks?

### The Effect of Positive Emotions on Entrepreneurial Intention

From the research results, positive emotions do not directly affect entrepreneurial intention, and entrepreneurial cognition as a mediating variable has an indirect effect. Busenitz and Barney (1997) found that entrepreneurs have a higher tendency to overconfidence than managers, and positive emotions can help entrepreneurs make quick decisions to save time and cognition resources. To a certain extent, positive emotions mean poor thoughts and irrationality and sometimes lead to decision-making errors. Whether college students choose employment or entrepreneurship in the future is an important decision and a major issue for college students in their life stages. They often make decisions after careful consideration rather than relying solely on enthusiasm and pride. Therefore, positive emotions will promote college students to enhance their entrepreneurial cognition and thus their entrepreneurial intention. The sample of this survey is a group of undergraduate participating in entrepreneurial competitions who are keener on entrepreneurship than ordinary students and are willing to practice. They often combine irrational and rational factors when making entrepreneurial decisions, rather than just relying on enthusiasm.

## Conclusion and Recommendations

This study investigated the relationship between positive emotions, entrepreneurial cognition (including arrangement scripts, willing scripts, and ability scripts), and entrepreneurial intention, established a structural equation model, and verified the influence mechanism among positive emotions and entrepreneurial cognition and entrepreneurial intention. In other words, positive emotions positively affect the arrangement scripts, willing scripts, and ability scripts of entrepreneurial cognition, and the arrangement scripts, willing scripts, and ability scripts of entrepreneurial cognition positively affect entrepreneurial intention. Furthermore, arrangement scripts and ability scripts fully mediate between positive emotions and entrepreneurial intention.

Positive emotions can help people improve their entrepreneurial cognition, which helps to improve people’s entrepreneurial intention. Therefore, we can make efforts in the following aspects of entrepreneurship education for youth and college students:

First of all, from the perspective of the government and society, the government and all sectors of society can not only provide youth and college students with free or low-cost entrepreneurship training to improve their entrepreneurial awareness and entrepreneurial skills, but also provide them with entrepreneurial support projects by giving a certain percentage of subsidies economically to enhance their entrepreneurial enthusiasm and willingness. Furthermore, it can also create and provide a good environment for youth and college students to start their own businesses, such as a maker space, to provide entrepreneurs and potential entrepreneurs with a comfortable space for working, social and resource sharing. Additionally, in terms of finance, low-interest or interest-free financial loan services can also be supported to young people and college students.

Second, from the perspective of colleges and universities, the direct source of college students’ entrepreneurial knowledge is the education of colleges and universities. Colleges and universities can provide opportunities for college students to learn entrepreneurial knowledge and provide a platform for some college students who are ready for entrepreneurship. The entrepreneurship education provided by schools can effectively improve the entrepreneurship cognition learning of college students and their entrepreneurship evaluation. First and foremost, schools should strengthen entrepreneurship education for college students. At present, China has provided a series of convenient conditions and policies for college students to start their businesses, and college entrepreneurship education plays a vital role in college students’ entrepreneurship. Therefore, colleges and universities should actively provide students with abundant entrepreneurial education resources. Such as business nurseries, business incubation base, they are setting up different types of entrepreneurship courses. Furthermore, we should look for people who have specific entrepreneurial experiences and have achieved success in society to make speeches to stimulate the sense of achievement of college students. Moreover, the school also should pay attention to students’ entrepreneurship practice. A business-related seminar and training for the students to actively organize the student to carry on the corresponding business planning, promote college students’ entrepreneurial typical successful cases, encourage students to actively participate in regional and national entrepreneurship competitions, and provide consultation and service for students with entrepreneurial intention. We should actively cultivate college students’ entrepreneurial cognition to promote their entrepreneurial intention.

Third, from the student’s point of view. Foremost, college students are supposed to actively learn about the entrepreneurship policies issued by the country, seek and understand the relevant knowledge of entrepreneurship, increase the knowledge reserve, timely update their cognition and views on entrepreneurship, and analyze the feasibility of entrepreneurship. Then form a self-perception of entrepreneurship through entrepreneurial practices to determine entrepreneurial ideas. What is more, college students have to improve their abilities. They should constantly learn knowledge and professional skills and actively participate in various entrepreneurial skills competitions to improve their innovation ability; college students should exercise their ability to identify market demand, judge entrepreneurial opportunities, and find their future entrepreneurial direction. College students should view the failure in entrepreneurship correctly and face it with a positive attitude. They should participate in entrepreneurial practice to exercise their ability to work under pressure and deal with problems, which helps them fully prepare for entrepreneurship.

## Limitations and Development

First of all, this study sampled undergraduates who had participated in innovation and entrepreneurship competitions, and this group first had some cognition of entrepreneurship and was keen on it. Therefore, the survey object is not for all college students, which has its limitations. In the future, if sampling is conducted for a larger scale of society, the research conclusions will be more representative. Second, the entrepreneurial emotions in this study are positive. However, entrepreneurial emotions also include negative. Therefore, combining positive emotions and negative emotions will better reflect the influence of entrepreneurial emotions. Third, this research is only aimed at Chinese college students; China’s current economic development and national policies provide college students with a suitable environment for innovation and entrepreneurship. So what are the youth and college students’ entrepreneurial intentions, different economic development stages, and different policy backgrounds? This is also a direction of future research.

## Data Availability Statement

The raw data supporting the conclusions of this article will be made available by the authors, without undue reservation.

## Ethics Statement

Ethical review and approval were not required for the study on human participants in accordance with the local legislation and institutional requirements. Written informed consent for participation was not required for this study in accordance with the national legislation and the institutional requirements.

## Author Contributions

B-SC contributed to writing–original draft. C-HY and BY contributed to writing–review and editing. X-ZW revised and supervised the entire work. All authors contributed to the article and approved the submitted version.

## Funding

This work was supported by Guangdong Province Education Science “13th Five-Year” Planning Project (2020GXJK420,2021GXJK484), Guangdong Province Philosophy and Social Science Planning Project (GD18XJY01), University of Electronic Science and Technology of China, Zhongshan Institute (CG201808 and JY202016), Humanities and Social Science Foundation of the Ministry of Education of China (19YJC630185), and the Foundation of Educational Commission of Guangdong Province of China (2018WTSCX199).

## Conflict of Interest

The authors declare that the research was conducted in the absence of any commercial or financial relationships that could be construed as a potential conflict of interest.

## Publisher’s Note

All claims expressed in this article are solely those of the authors and do not necessarily represent those of their affiliated organizations, or those of the publisher, the editors and the reviewers. Any product that may be evaluated in this article, or claim that may be made by its manufacturer, is not guaranteed or endorsed by the publisher.
